# Leukocytosis Associated with Clozapine Treatment: A Case Series and Systematic Review of the Literature

**DOI:** 10.3390/medicina57080816

**Published:** 2021-08-11

**Authors:** Pasquale Paribello, Mirko Manchia, Massimo Zedda, Federica Pinna, Bernardo Carpiniello

**Affiliations:** 1 Section of Psychiatry, Department of Medical Sciences and Public Health, University of Cagliari, 09124 Cagliari, Italy; pasqualeparibello@gmail.com (P.P.); mirkomanchia@unica.it (M.M.); massimo_zedda@icloud.com (M.Z.); bcarpini@iol.it (B.C.); 2 Unit of Clinical Psychiatry, University Hospital Agency of Cagliari, 09124 Cagliari, Italy; 3 Department of Pharmacology, Dalhousie University, Halifax, NS B3H 4R2, Canada

**Keywords:** clozapine, atypical antipsychotics, neutrophilia, leukocytosis, systematic review

## Abstract

Background and Objectives: Clozapine is the only antipsychotic approved for treatment-resistant schizophrenia. Despite its superior efficacy profile as compared with other antipsychotics, clozapine remains underutilized. Clozapine monitoring systems clearly describe the proposed management of clozapine-induced neutropenia; however, no specific mention is made of how to interpret neutrophilic leukocytosis, despite that being a relatively frequent finding. Prescribers unfamiliar with this molecule may misjudge its clinical significance, potentially leading to untimely treatment interruption. Here, we systematically review the literature on the risk of neutrophilic leukocytosis during clozapine treatment, and describe eight additional cases among our patient cohort. Materials and Methods: We performed a systematic review of the literature on PubMed and Embase using the PRISMA 2020 guidelines, and selected all original reports describing either (1) the prevalence of neutrophilic leukocytosis during clozapine treatment, or (2) the clinical significance of neutrophilic leukocytosis. We described eight additional cases of neutrophilic leukocytosis during clozapine treatment while attending an outpatient psychiatric clinic. Results: Our research ultimately yielded the selection of 13 articles included in this systematic review. The case series highlighted the presence of stable and clinically unremarkable neutrophilia during a follow-up ranging from one to ten years. Conclusions: Existing evidence indicates that leukocytosis associated with clozapine treatment can be considered as an asymptomatic and benign condition, suggesting that no change in clozapine treatment is needed upon its detection.

## 1. Introduction

Schizophrenia (SCZ) is a severe psychiatric disorder affecting approximately 1% of the general population [1]. Although the past twenty years have seen the development of antipsychotics with innovative mechanisms of action, namely partial agonism at D_2_ and 5-HT_1_ receptors, there is a large proportion of patients affected by SCZ who present suboptimal response or treatment resistance. For this population of patients, clozapine remains the most effective therapeutic option. Indeed, currently, clozapine is the only approved drug for treatment-resistant SCZ [2]. In SCZ clozapine has also shown a superior profile of efficacy for the treatment of suicidality [3], co-morbid substance use disorder [4], hostility [5,6,7], psychosis in Parkinson disease [8], and treatment-refractory mania [9]. Despite its known impact on metabolism [10], several lines of evidence suggest a positive effect on overall survival as compared to no treatment or to other antipsychotics [11,12,13,14]. Despite the mounting evidence suggesting its value in the management of the most severe cases of SCZ, clozapine remains significantly underutilized [15]. One possible explanation is the perception from prescribing physicians as a drug that needs too extensive clinical monitoring and that presents an unfavorable safety profile. Thus, experts have tried to increase the knowledge and confidence in the management of common side effects as well as in the interpretation of possible blood markers derangements of clozapine [16]. Thirty years have passed since the reintroduction of clozapine in the market following the influential paper authored by Kane [17] with mandatory blood monitoring required in numerous countries. Arguably, this factor may have further reduced the likelihood of using clozapine in certain settings but helped to determine with a reasonable level of confidence the real incidence of severe hematological side effects, up to the point of questioning the usefulness of these protocols [18]. However, considering the complex history surrounding this drug, determining the clinical significance of either a leukopenia or a leukocytosis represents a particularly critical step. In most laboratories, the reference range for circulating leukocytes is included between 4500 and 11,000 units per mm^3^ (units/mm^3^). Under physiological conditions, neutrophils represent their vast majority, ranging from 1800 up to 7700 units/mm^3^ [19]. When considering the possible underlying causes for these anomalies, clinicians should always evaluate the absolute count of each leukocyte subpopulation: the total leukocyte counts by themselves can be misleading as an absolute leukocyte count falling within the normal range may instead be harboring an abnormal composition in one or more of its subpopulations. Clozapine monitoring systems clearly describe the proposed management of clozapine-induced neutropenia. However, no specific mention is made of how to interpret neutrophilic leukocytosis, despite it being a relatively frequent finding. Prescribers unfamiliar with this molecule may misjudge its clinical significance, potentially leading to untimely treatment interruption. In the present paper, we offer the reader a general overview of the clinical importance of this clinical finding by systematically reviewing the pertinent literature, as well as describing eight additional cases identified within our patient cohort.

## 2. Materials and Methods

### 2.1. Search Strategy

The following systematic review has been performed according to the Preferred Reporting Items for Systematic Reviews and Meta-Analysis 2020 (PRISMA)[20]. A database search was performed on Medline and Embase up to the 26th of April 2021. The search strategy employed included the following terms: “clozapine” OR “clozaril” OR “leponex” OR “clopine” AND “leukocytosis” OR “neutrophilia” OR “blood dyscrasia” OR “white blood cells”. We augmented this search strategy by tracking citations of the reference list of the identified articles.

### 2.2. Eligibility Criteria

Study selection involved the analysis of the title and the abstract to identify papers relevant to this review. After this first screening, the full version of the corresponding paper was reviewed: we included all original reports describing either (1) the prevalence of neutrophilic leukocytosis during clozapine treatment or (2) the clinical significance of such a finding. The selected papers were included after a consensus of two independent reviewers was reached. Conference abstracts, case reports, case series and cohort studies with reports available in English were also assessed.

### 2.3. Data Extraction

All included studies were qualitatively examined by two investigators (PP and MZ). We extracted data related to: demographics, study design, eventual medical co-morbidities, treatment duration, concomitant pharmacotherapy, smoking status, diagnosis, clinical outcome and leukocyte count range when available.

### 2.4. Clinical Characterization

We extracted data though an accurate retrospective assessment of the longitudinal clinical history of these patients attending an outpatient psychiatric clinic, the Section of Psychiatry of the Department of Medical Science and Public Health, University of Cagliari, Cagliari, Italy. This is a community mental health center operating in a catchment area of more than 80,000 people, with approximately 2500 patients regularly followed up. We identified 74 patients affected by SCZ and schizoaffective disorder who were treated with clozapine at the time of data collection. Of these, all patients gave written and verbal consent to allow reanalysis of clinical data for research purposes in an anonymized form.

## 3. Results

### 3.1. Results of the Systematic Search

**Figure 1:**
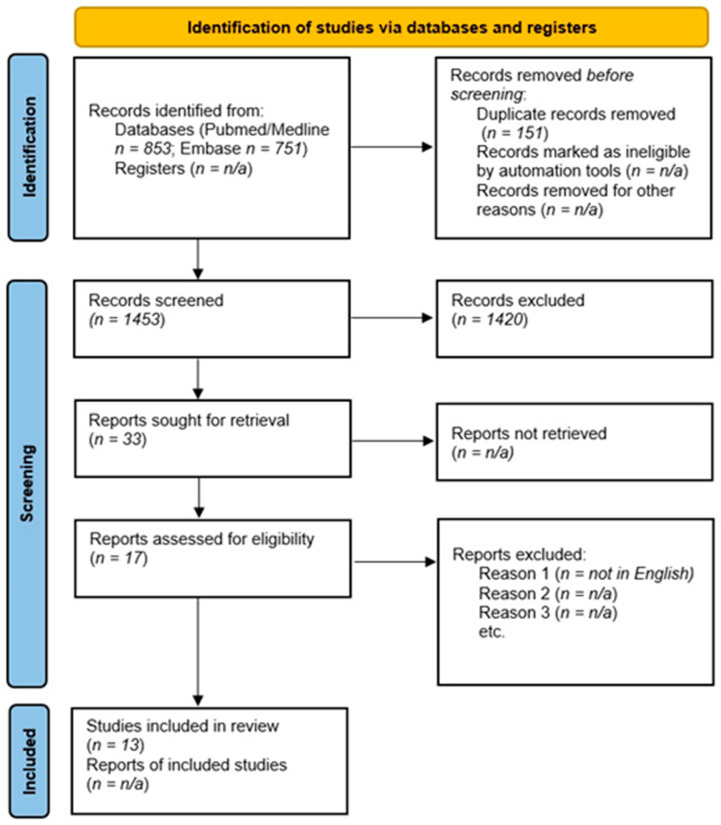
PRISMA flow-chart of the systematic review on leukocytosis during clozapine treatment.

A total of 1453 articles (PubMed/Medline = 853; Embase = 751) was found after excluding duplicates (*n* = 151), leading to the selection of 33 articles with an abstract and/or title pertinent to our research (Figure 1). After assessing the corresponding full article, we selected 13 papers to be included in this review. One article was excluded because it was not written in English. A summary of the findings of the included studies is provided in Table 1.

### 3.2. Case Series

In the following section, we report on eight additional neutrophilia cases selected from our own cohort of 74 patients on clozapine treatment (Table 2), corresponding to an unadjusted rate of 10.8%.

#### 3.2.1. Case 1

A 47-year-old Caucasian male affected by schizoaffective disorder and mild intellectual disability developed persistent leukocytosis lasting over a 6-year period under treatment with clozapine and lithium. His medical history was otherwise unremarkable.

#### 3.2.2. Case 2

A 44-year-old Caucasian male affected by SCZ presented wide fluctuations in his leukocyte counts documented over a seven-year period. The medical history was significant for co-morbid chronic obstructive pulmonary disease (COPD) and diabetes mellitus type 2 (DMT 2). Apart from clozapine, his pharmacotherapy comprised atenolol, atorvastatin and alprazolam.

#### 3.2.3. Case 3

A 47-year-old Caucasian male with a schizoaffective disorder diagnosis presented consistently elevated leukocyte counts over a three-year period. Along with clozapine, his pharmacotherapy comprised oxcarbazepine, biperiden, promazine, delorazepam and clonazepam. His medical history was relevant for concomitant use of various substances of abuse (e.g., cocaine, amphetamine, heroin), and chronic Hepatitis C Virus (HCV) infection, although no additional information was available regarding the activity of the infection.

#### 3.2.4. Case 4

A 37-year-old Caucasian female affected by schizoaffective disorder presented wide fluctuations in the leukocyte levels over a 4-year period. The past medical history is significant for hypothyroidism treated with hormonal replacement therapy and previous voluntary termination of pregnancy. Her pharmacotherapy comprised clozapine, levothyroxine, gabapentin, aripiprazole, delorazepam and lorazepam.

#### 3.2.5. Case 5

A 47-year-old Caucasian male affected by schizoaffective disorder presented persistently elevated leukocyte counts over a one-year period. His past medical history was relevant for poliomyelitis, celiac disease, and beta-thalassemia trait. His pharmacotherapy included paliperidone, escitalopram, delorazepam, lamotrigine.

#### 3.2.6. Case 6

A 57-year-old Caucasian female with a diagnosis of SCZ and mild intellectual disability presented frequent fluctuations in the leukocyte counts, with values ranging from normal to moderately elevated. Her past medical history was significant for a diagnosis of epilepsy. Along with clozapine, her pharmacotherapy comprised gabapentin, fenobarbital, biperidene, risperidone and clonazepam.

#### 3.2.7. Case 7

A 49-year-old Caucasian female with a diagnosis of schizoaffective disorder presented significant fluctuations in her leukocyte levels during the last ten years. Her medical history was significant for alfa-thalassemia, and consistent with this diagnosis, her blood tests revealed chronic anemia alongside the leukocytosis. Along with clozapine, her pharmacotherapy comprised delorazepam and lithium.

#### 3.2.8. Case 8

A 51-year-old Caucasian male affected by SCZ, presented wide fluctuations in leukocytes over a 5-year period on clozapine therapy. Along with clozapine, his pharmacotherapy comprised zuclopenthixol, haloperidol, flurazepam, gabapentin, choline.

## 4. Discussion

Notwithstanding the significant efficacy of clozapine, in the years following its re-introduction in clinical use numerous side-effects have been described, some relatively minor and others potentially life-threatening. Its use is associated with an increased risk of weight gain, metabolic syndrome, sialorrhea, constipation, sedation, enuresis and seizures. Despite the great attention devoted to the possible development of agranulocytosis (or severe neutropenia as it is currently described, i.e., < 500 neutrophils per microliter), this is a rare event: it is estimated that the risk of observing a fatal agranulocytosis case during regular monitoring is 1 in 8000 and with a negligible risk after the first 12 months of treatment. This is a much lower risk as compared with the risk of fatal myocarditis or fatal pulmonary embolism associated with its use, estimated as 1 in 4500 and 1 in 1000, respectively [33]. Prescribers and service users alike need to be particularly attentive and proactive in monitoring for the emergence of these major side effects. However, despite these findings, clozapine remains one of the most efficacious pharmacological treatments, and the one associated with the lowest risk of overall-cause death and treatment discontinuation as compared with the absence of treatment and with the other available treatments [14,34]. This is a particularly striking finding, especially considering how a long-acting injectable version of this medication is not available, and how its use is limited to the most severe, treatment-resistant cases.

According to the available data, clozapine use is associated with a benign and transient leukocytosis [35,36], the incidence of which ranges from 0.6% to 7.7% [29,37]. A more persistent form has been described mainly in case reports and especially with concomitant lithium use [38]. More recently, Fabrazzo et al. reported leukocytosis in 37.8% of a clozapine-treated cohort of patients, with incidence rates of 11.1% and 26.7% for the transient and persistent types, respectively [22]. Male gender [27,28,29,30,32,38], and lithium co-administration represent the most important risk factors for leukocytosis [39,40]. A retrospective one-year study described a 48.9% cumulative incidence for neutrophilia in a cohort comprising 101 patients treated with clozapine [38]. More recently, a retrospective study described hematological side effects in a 303 patients from India during a 49-month period. About one-fifth of the total cohort (64 individuals) developed hematological anomalies, but no neutrophilia case was described, in contrast with previous reports [41]. Further research is needed to clarify the possible contribution of ethnic factors in the observed incidence of blood dyscrasia.

Smoking is another recognized cause of idiopathic leukocytosis regardless of pharmacotherapy [19], with some studies reporting an association between smoking cessation and leukocyte count reduction [42]. Smoking is also a clinically significant inducer of the CYP450 isoenzyme primarily responsible for clozapine metabolism (i.e., CYP1A2), with smokers needing nearly 1.5 times higher clozapine doses as compared with non-smokers [43]. This effect is independent of nicotine and results from the interactions of hydrocarbons typically found in smoke and cytosolic transcription factors, yielding increased CYP1A2 gene transcription [44,45,46,47]. Similarly, lithium-induced neutrophilia is a well-known hematological effect [19] resulting from a complex interaction with the bone marrow, promoting an increased peripheral neutrophilic count [48]. Although it does not appear to prevent severe neutropenia (i.e., <500 neutrophils/mm^3^), lithium augmentation is advised to prevent recurrent clozapine discontinuations in those individuals predisposed to neutropenia with neutrophil counts falling below the danger threshold indicated by the local clozapine monitoring guidelines [49,50].

As with clozapine-induced neutropenia, the precise mechanism underlying neutrophilia is currently unknown; however, a variety of different hypotheses have been proposed to explain the interaction between clozapine and the hematopoietic system’s cells. The most popular among them regards this as a dichotomic process, implying both direct and indirect effects with opposing results. According to this theory, clozapine directly induce an increased production of reactive oxygen species resulting in a higher expression of pro-apoptotic genes, such as p53, Bax-α and Bik. Increased release of cytokines such as TNF-α, IL-2, IL-6, and G-CSF instead indirectly induces the expression of anti-apoptotic proteins, capable of promoting the differentiation and maturation of myelocytes. In this setting, leukocytosis ensues from the preponderance of anti-apoptotic factors, tipping the scale in favor of an increased total leukocyte count, and in particular in the neutrophil count [25,51,52]. An additional hypothesis involves the inflammatory response mediated by clozapine itself on blood cells, which appears particularly evident during the first month of treatment [25,53].

The increase in the absolute neutrophil counts appears directly proportional to higher clozapine doses, suggesting a dose-dependent drug effect. Moreover, a downward dose titration tends to produce a normalization in the neutrophil counts, offering further support to this notion [21,25,27]. Mauri et al. reported an association between clozapine metabolism and blood dyscrasias, with an increased norclozapine/clozapine ratio being significantly associated with a reduction in the neutrophil count [54]. On the other hand, Centorrino et al. reported no correlation between the norclozapine/clozapine ratio and decrements in leukocytes and granulocytes. Interestingly, in the latter paper, no specific distinction was made between the granulocyte count and the neutrophilic count [55].

A further case series [28] described chronic clozapine-induced leukocytosis in seven individuals. None of them presented medical comorbidities (e.g., traumas, burns) that could contribute to the increased leukocyte count, although all of them were smokers. The highest leukocyte count reported was 19,800 units/mm^3^, with durations ranging from two to five years. No adverse consequence was reported, underscoring the benign nature of this phenomenon. Popli reported an additional clozapine-induced leukocytosis case in a 50-year- old male affected by schizophrenia. From the 16th to the 23rd weeks of treatment, cyclic variations in the leukocyte counts were observed with values ranging from normal to significant elevations [30].

One case report described a 37-year-old Caucasian male affected by refractory schizophrenia developing chronic leukocytosis on clozapine. During a two-year period, his leukocyte count fluctuated from a minimum of 10,000 units/mm^3^ to a maximum of 28,000 units/mm^3^ [27]. Capllonch and coauthors reported on a cohort of 55 women treated with clozapine presenting higher leukocyte and neutrophil levels after the first three to four weeks of treatment, with persistent elevations from the baseline observed during the 18-week follow-up [51]. In a case series, Prisco et al. proposed neutrophilic leukocytosis as a possible predictor of resistance to clozapine treatment [56], in accordance with previous reports [57]. Two individuals affected by treatment-resistant SCZ, previously stable, experienced a decompensation in their clinical condition concomitant with the onset of the leukocytosis. Before this event, no blood dyscrasia was observed in either of the two subjects. An increased leukocyte count was observed with increasing daily doses, while subsequent dose reductions and the following withdrawal were associated with leukocyte count normalization in both cases, further supporting the causative role of clozapine in inducing leukocytosis [56]. Regarding the putative association between neutrophilia and the clinical outcome, Fabrazzo et al. [22] found persistent neutrophilia associated with a loss of clozapine efficacy over time in their cohort. At present, the hypothesized association between leukocytosis and clinical worsening remains speculative at best, with the former representing a benign and transient phenomenon. Once diagnosed, after ruling out possible alternative causes, clozapine-induced leukocytosis should never warrant treatment discontinuation, not even temporarily [23,49].

It remains unclear whether a particular diagnostic category may be associated with a higher risk of neutrophilia during clozapine treatment. For a variety of selected studies, there was no mention of the set of diagnostic criteria applied. When specified, a clear description of the relative frequency of each nosological condition was not consistently indicated. Similarly, smoking status and age of onset for the primary diagnosis were not consistently reported. In our cohort, five patients were diagnosed with SZA disorder according to the DSM-IV criteria, however the gap in the literature renders difficult the comparison of our data with previous evidence. In addition, our patients’ group includes mostly male individuals, in line with previous reports signaling a possible increased risk among men. Smoking appears to be a commonly co-occurring finding, and in the papers selected for this review where smoking status is available, it is present in nearly half of the samples. This is consistent with the observations in our patient cohort of six out of eight individuals reported smoking regularly. Finally, the median age of onset of the main psychiatric diagnosis was 19 years old (mean 21.1), but it is not currently possible to establish the significance of this finding.

### Limitations

The findings of this study should be considered in light of a series of limitations. First, the systematic search highlighted the presence of sparse literature, with some papers reporting only the total leukocyte counts rendering their interpretation more difficult. However, neutrophilic leukocytosis is assumed to be the prevailing mechanism in the presented cases. In addition, for a significant number of studies, it was not possible to ascertain a clear causal link with clozapine use, as there were other concomitant factors (e.g., smoking). A third limitation concerns the retrospective assessment of data regarding the clinical course of the patients included in this case series which might have impacted the accuracy of the recollection of data, particularly those related to overlapping treatments and/or medical conditions or smoking. It should be noted, however, that clinical information was collected during longitudinal prospective systematic follow-ups, and as such, was less prone to recall bias. Finally, the small sample size did not allow a quantitative analysis but only for narrative description of cases.

## 5. Conclusions

Existing evidence indicates that leukocytosis associated with clozapine treatment is an asymptomatic and benign condition. We also reported on eight additional individuals presenting stable and persistent leukocytosis during follow-up ranging from one to ten years, further expanding the available evidence regarding this common laboratory finding during clozapine treatment. Considering these data, no change in clozapine treatment is therefore needed upon its detection. The cases presented in this paper add to the pre-existing body of evidence, showing that safe management of asymptomatic clozapine-induced leukocytosis is feasible, particularly when other possible etiologies are ruled out applying accurate clinical monitoring.

## Figures and Tables

**Table 1:** Articles retrieved with the systematic review.

References (year)	Study Design	Leukocyte (and *Neutrophil*) Levels in Units/mm^3^	Treatment Duration on Clozapine	Concomitant Pharmacological Treatment Other than Clozapine	Presence of Medical Comorbidities	Demographic Data, Psychiatric Diagnosis, (*Clinical Outcome*)
[21]	Case report	22,100; (17,680)	25 days	Lorazepam	Not applicable	48 y.o. F; SCZ; (*not applicable*)
[22]	Retrospective cohort study	>15,000; (>7000)	18 weeks; neutrophilia was observed in 37.8% of the total cohort comprising 145 clozapine-treated individuals	63 patients received benzodiazepines, 33 mood stabilizers, and 9 antidepressants (concomitant treatment with mood stabilizers or benzodiazepines was associated with transient anemia; co-treatment with antidepressants was associated with transient eosinophilia)	The presence of medical comorbidities did not represent a significant risk factor for the development of neutrophilia	135 individuals; 70 M, 65 F; 45.1 y.o. among M; 37.9 y.o. among F; 66/135 smokers; 125 SCS, 10 BPS; (*persistent neutrophilia was associated with a tendency to lose efficacy over time*)
[23]	Case report	24,300; (not available)	25 days	Not available	No relevant comorbidity was found despite a broad medical evaluation	41 y.o. F; SCZ; (*not applicable*)
[24]	Retrospective chart review study	(>7500)	One-year study; neutrophilia developed after a median of 6.5 weeks on clozapine; 48.9% cumulative incidence for neutrophilia; neutrophilia preceded neutropenia in three cases of a total of five neutropenia cases	42/101 individuals on clozapine monotherapy (no significant differences in the development of blood dyscrasias were found with polytherapy vs. clozapine monotherapy)	33 individuals presented medical comorbidities (11 D.M., 10 HLP, 4 HOT, 7 HTN); no data regarding concurrent infections during the one-year period	101 individuals; mean age 35–71 y.o.; 74 M, 27 F; 55 smokers; 80 SCZ; 19 SCA; 1 BD; 1 DD; (*not applicable*)
[25]	Case report	22,000; (18,200)	12 weeks	Not applicable	Not applicable	51 y.o. M; SCA; (*not applicable*)
[26]	Case series	First case: up to a maximum of 14,600 (10,500); second case: 19,400 (not available)	First case: starting from 45th week through to 59th week;second case: over an 18-month follow-up	Lithium in both cases	Not applicable	Two M individuals; 30 y.o. and 56 y.o.; two SCA; (*not applicable*)
[27]	Case report	99% of leukocyte counts in a three-year period were >11,000; (not applicable)	Three years	Fluoxetine, Clonazepam, Disulfiram, Esomeprazole, Clomipramine	No relevant comorbidity was found despite a broad medical evaluation	37 y.o.; M; SCZ
[28]	Case series	>11,000 (available only for 2/7 cases, >7800)	Two to eight years	Clonazepam, Olanzapine, Quetiapine, Valproic Acid (3/7 on clozapine monotherapy)	BPH; CAD; CHF; DM; GERD; HLP; HTN; HON; SD	Seven M individuals, all smokers; age range 42–52 y.o.; six SCZ, one of those with M.R. comorbidity; one SCA; (*not available*)
[29]	Retrospective cohort study	15,000–21,000; among 2404 included patients, 185 individuals presented leukocytosis, with a 7% incidence	Four years	Not available	Not available	1515 M; 889 F; SCZ; (*no significant impact of the leukocytosis on the medical or psychiatric prognosis; it resolved spontaneously in all patients*)
[30]	Case report	Intermittent leukocytosis from the 15th through the 24th week of treatment up to 15,000 (not available)	23 weeks	Atenolol	Head injury; HTN; splenectomy	50 y.o. M; SCZ; non-smoker; (*not applicable*)
[31]	Retrospective chart review study	>11,000	Transient (only in one case it lasted for two years)	No differences in blood dyscrasia incidence were found between clozapine monotherapy vs. polypharmacy		68 individuals; 28.9 y.o. among males; 34.2 y.o. among females; 43% developed neutrophilia; one individual presented chronic leukocytosis; (*not applicable*)
[32]	Case report	Observed after 44 weeks of treatment through week 54 and up to a maximum of 16,000 (>7800)	54-week follow-up	Benzodiazepines, Amitriptyline	Mild pharyngeal irritation with slight swollen cervical glands; despite a broad medical evaluation, no relevant comorbidity was found	55 y.o. M; smoker; PDM; (*worsening depressive symptoms*)

Abbreviations: BPD—bipolar disorder; BPH—benign prostatic hypertrophy; BPS—bipolar spectrum; CAD—coronary artery disease; CHF—congestive heart failure; CLL—cellulitis; DD—delusional disorder; DM—diabetes mellitus; F—female; GERD—gastroesophageal reflux disorder; HLP—hyperlipidemia; HON—hyponatremia; HOT—hypothyroidism; HTN—hypertension; M—male; MR—mental retardation; PMD—psychotic major depression; PUD—peptic ulcer disease; SCS—schizophrenia spectrum; SCZ—schizophrenia; SCA—schizoaffective; SD—seizure disorder; y.o.—years old.

**Table 2:** Main clinical and treatment-related characteristics of reported cases of leukocytosis during clozapine.

Case #, Sex, Age	Leukocytes and Neutrophils Range (Units/mm^3^)	Pharmacotherapy (Clozapine Dose mg/day)	Psychiatric Diagnosis, and Medical/Psychiatric Comorbidity	Clozapine and Norclozapine Plasma Levels (ng/mL)	Clozapine/Norclozapine Ratio	Follow-Up Duration (years)	Smoking Status (Cigarettes/Day)
1, M, 47	11,000–15,000;	Clozapine (400), lithium	SCA, MID	404, 313	1.29	6	10
2, M, 44	9900–16,000; 6100–12,000	Clozapine (400), atenolol, atorvastatin, alprazolam	SCZ, COPD, DMT 2	475, 329	1.44	7	20
3, M, 47	11,800–14,600; 5300–7900	Clozapine (300), oxcarbazepine, biperiden, promazine, delorazepam, clonazepam	SCA, HCV, SUD	580, 480	1.2	3	20
4, F, 37	7600–21,600; 5600–17,600	Clozapine (225), levothyroxine, gabapentin, aripiprazole, delorazepam, lorazepam	SCA	451, 380	1.18	4	40
5, M, 47	7500–20,450; 5200–20,450	Clozapine (175), paliperidone, escitalopram, delorazepam, lamotrigine	SCA, CD, BTT	109, 69.4	1.57	1	20
6, M, 57	5500–14,800; 2800–11,700	Clozapine (225), gabapentin, phenobarbital, biperiden, risperidone, clonazepam	SCZ, MID	290, 223	1.3	3	None
7, F, 49	10,500–13,300	Clozapine (350), lithium	SCA, ATT	443, 348	1.27	10	None
8, M, 51	10,500–24,000; 6700–18,000	Clozapine (600), zuclopenthixol, haloperidol, flurazepam, gabapentin, choline	SCZ	353, 108	3.2	5	15

Abbreviations: ATT—alpha thalassemia trait; BTT—beta thalassemia trait; CD—celiac disease; COPD—chronic obstructive pulmonary disease; DMT 2—diabetes mellitus type 2; F—female; HCV—hepatitis C virus; M—male; MID—mild intellectual disability; SCA—schizoaffective disorder; SCZ—schizophrenia; SUD—substance use disorder; y.o.—years old.

## Data Availability

The data presented in this study are available on request from the corresponding author.
